# Stimulated Parotid Saliva Is a Better Method for Depression Prediction

**DOI:** 10.3390/biomedicines10092220

**Published:** 2022-09-07

**Authors:** Yangyang Cui, Hankun Zhang, Song Wang, Junzhe Lu, Jinmei He, Lanlan Liu, Weiqiang Liu

**Affiliations:** 1 Tsinghua Shenzhen International Graduate School, Tsinghua University, Shenzhen 518055, China; 2 Department of Mechanical Engineering, Tsinghua University, Beijing 100084, China; 3 Biomechanics and Biotechnology Lab, Research Institute of Tsinghua University in Shenzhen, Shenzhen 518057, China

**Keywords:** saliva, cortisol, biomarker, depression, prediction, level, collection, methods, correction, blood

## Abstract

Background: Saliva cortisol is considered to be a biomarker of depression prediction. However, saliva collection methods can affect the saliva cortisol level. Objective: This study aims to determine the ideal saliva collection method and explore the application value of saliva cortisol in depression prediction. Methods: 30 depressed patients and 30 healthy controls were instructed to collect saliva samples in the morning with six collection methods. Simultaneous venous blood was collected. Enzyme-linked immunosorbent assay was used to determine the cortisol level. The 24-observerrated Hamilton depression rating scale (HAMD-24) was used to assess the severity of depression. Results: The significant differences in saliva cortisol levels depend on the saliva collection methods. The level of unstimulated whole saliva cortisol was most correlated with blood (r = 0.91). The stimulated parotid saliva cortisol can better predict depression. The area under the curve was 0.89. In addition, the saliva cortisol level of the depression patients was significantly higher than the healthy controls. The correlation between the cortisol level and the HAMD-24 score was highly significant. The higher the saliva cortisol level, the higher the HAMD-24 score. Conclusions: All the above findings point to an exciting opportunity for non-invasive monitoring of cortisol through saliva.

## 1. Introduction

Depression is a mental illness characterized by a long period of sadness with several social and psychiatric factors that have been identified as the main cause of suicide [1,2]. Moreover, its lifetime prevalence rate is as high as 16% [3]. Despite the high prevalence and significant morbidity of depression in the population, the exact physical causes of depression remain unknown [4]. Some studies pointed out that the factors that should draw attention to the study of depression, especially related to depression and stress, may include, but are not limited to, the pathogenic involvement of diet and microbiota, stress and mitochondrial impairment, aging and comorbidity, and cognitive and motor function [5,6,7]. Among them, stress has been proved to be one of the underlying causes of depression [8]. Further research on the biological pathways related to stress in people with depression may help to understand the causes of stress related to depression [9,10].

The hypothalamic-pituitary-adrenal (HPA) axis is one of the potential neurobiological pathways of depression, and the HPA axis reflects the regulation of stress by the neuroendocrine system [11,12]. Cortisol is the main component of the glucocorticoid secreted by the adrenal cortex. Its level fluctuates with the circadian rhythm of the HPA axis. It can reflect the function of the HPA axis [13,14]. For most biotypes, cortisol levels are at their highest in the morning, which can reflect the function of the adrenal cortex, usually around 9 a.m. [15]. Saliva has been proven to have high correlation values to cortisol levels in blood with a non-invasive in situ collection method, so it is more lucrative for cortisol determination compared with blood [16,17]. In clinical practice, about 80% of the cortisol in the blood is combined with cortieosteroid-binding globulin (CBG), and the rest is in a free state [18]. It is only the free fraction that is biologically active and can activate signaling pathways via glucocorticoid hormone receptors in cells [19]. Since saliva does not contain CBG, saliva cortisol can well reflect the level of free cortisol with biological activity in the blood [20]. Moreover, saliva contains biomarkers, which, like blood, can reflect changes in human physiological functions. Thus, saliva can be an ideal alternative to blood [21]. Therefore, it has been widely used in mental and psychological research [22]. Previous studies have found that the saliva cortisol level of patients with depression is higher than that of healthy people [23,24], but some researchers hold the opposite conclusion [25,26] or believe that there is no difference [27]. In addition, a number of studies have shown that saliva sampling methods have an impact on the content of cortisol in saliva, and there is no parallel comparison between these saliva cortisol measurements and serum cortisol values [28,29].

So, in this study, participants included 30 depressed patients and 30 healthy controls who were instructed to collect saliva samples in the morning when waking up. They used six collection methods, including unstimulated and stimulated whole saliva (UWS, SWS), unstimulated and stimulated sublingual/submandibular saliva (USS, SSS), and unstimulated and stimulated parotid saliva (UPS, SPS). Simultaneous venous blood sampling was collected. Enzyme-linked immunosorbent assay was used to determine the level of cortisol. The 24-observer-rated Hamilton depression rating scale (HAMD-24) was used to assess the severity of depression in the study participants. The differences in saliva cortisol levels between depression patients and healthy controls were compared, and the relationship between the severity of depression and saliva cortisol levels was analyzed. Moreover, the value of saliva cortisol in the diagnosis of depression and the receiver operating characteristic (ROC) method in the diagnosis of depression were analyzed. 

## 2. Materials and Methods

### 2.1. Participants and Study Design

In this study, 60 participants were included, which were divided into two groups: the patient group (N = 30) and the control group (N = 30). The patient group meets the diagnostic criteria and meets the tenth edition «The International Statistical Classification of Diseases and Related Health Problems 10th Revision» (ICD-10) [30]; selection criteria: 18 to 65 years old with no history of psychotropic medication and diagnosed by two associate chief physicians. A total of 30 healthy controls from the physical examination center during the same period were selected as the control group. Selection criteria: 18 to 65 years old, regardless of sex. On the day of saliva collection, two psychiatrists assessed all participants with HAMD-24, and the total score of the scale reflects the severity of depression. A total score of <8 points, no depression; a total score of 8–20 points, may have mild depression; a total score of 20–35 points, mild to moderate depression; total score >35 points, severe depression [31]. All study participants signed an informed consent form. The collection of human blood and saliva samples was approved by the local ethics committee at Tsinghua University.

### 2.2. Laboratory Tests

**Figure 1:**
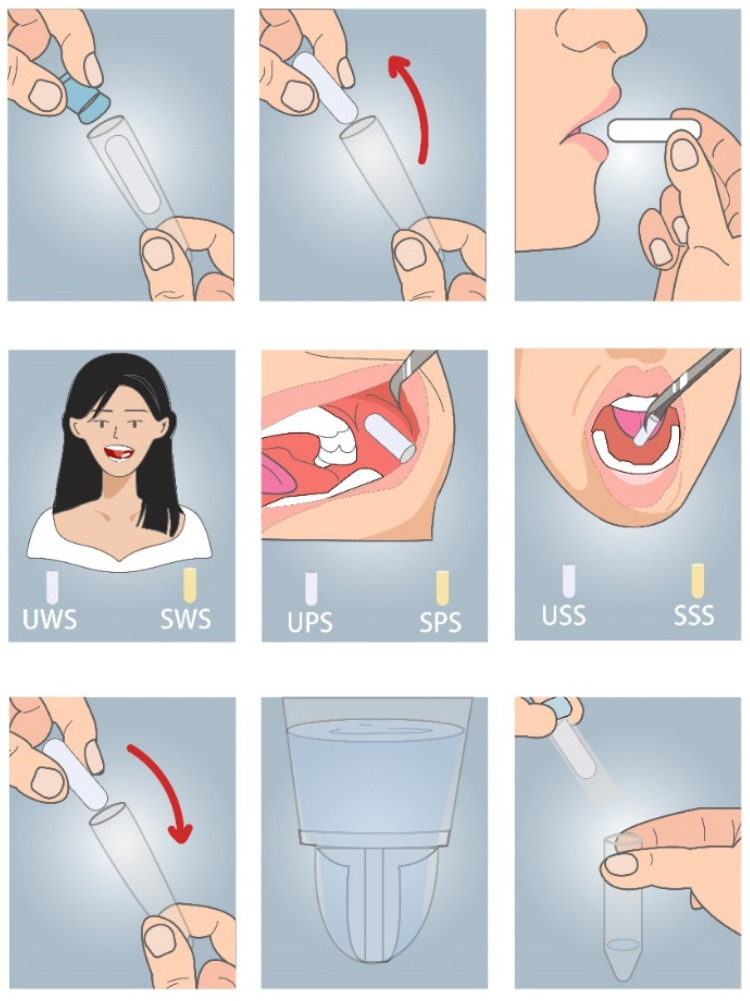
Six methods for collecting saliva samples, UWS/SWS: the swab in the test tube was taken out and put in the mouth to chew for 3 min; UPS/SPS: the swab was placed near the left parotid duct, and it was taken out after 3 min; USS/SSS: the swab was put under the tongue, and it was taken out after 3 min.

Before the collection, the participants were told to pay close attention to the collection: no drinking within 12 h before the collection, no eating within 1 h, and no brushing or drinking water within 10 min. The collection time was 7:30–9:30 in the morning. For each participant, samples of the parotid gland, mandibular/sublingual gland, and whole saliva were collected with and without stimulation (as shown in Figure 1). The swabs included acid stimulated and untreated swabs, so the different swabs in the parotid, sublingual/submandibular, and whole mouth were represented by UWS, SWS, UPS, SPS, USS, and SSS, respectively, where the saliva collection method was the same as in our previous studies [32,33]. All the saliva samples were collected in the same clinical room and at the same time, between 7:30 and 9:30 in the morning. To prevent the degradation of sensitive peptides, all samples were collected in prechilled polypropylene tubes on ice. The total amount of saliva collected by all methods is 5 mL. In the end, it was routinely transported to the laboratory, transferred to a centrifuge tube, centrifuged, and the supernatant was taken and stored at −20 °C for later use. After the last saliva sample was collected, venous blood samples were collected from all participants. The sample was gently mixed for 1 min and then immediately placed on ice for 30 min. The sample was then centrifuged at 1000 r for 15 min at 4 °C, and the upper 2/3 aliquot of plasma was stored at −80 °C until analysis. Saliva cortisol levels were determined using a particular enzyme-linked immunosorbent test (ELISA, Beijing Furui Runkang Biotechnology Co., Ltd., Beijing, China).

### 2.3. Statistical Analyses

Statistical analyses were carried out using GraphPad Prism 8.0 (GraphPad Software, San Diego, CA, USA, www.graphpad.com). In order to facilitate comparisons between groups, the data χ^2^ were reported as relative numbers. Measurement data were transformed into a normal distribution and presented as mean standard deviation ($\bar{x}$ ± s), and a t-test was employed to make group comparisons. To determine if the data are normally distributed, we utilized the Shapiro–Wilk test. When describing data that were not normally distributed, minimum and maximum values were used, whereas when describing data that was regularly distributed, the standard normal distribution statistic, ($\bar{x}$ ± s), was used. Because the saliva cortisol level of the control group was normally distributed while the saliva cortisol level of the patient group was non-normally distributed, the saliva cortisol level of the two groups was compared using the Wilcoxon rank sum test. The correlation between saliva cortisol and HAMD-24 score was analyzed by Spearman rank correlation analysis. The saliva cortisol of the participants of different genders and ages in each group was compared using a t-test of two independent samples. In this study, the ROC method was used to comprehensively evaluate the diagnostic value of saliva cortisol testing for depression. *p* < 0.05 indicated that the difference was statistically significant.

## 3. Results

### 3.1. Sample Characteristics

The 30 participants in the depression group included 12 males (40%) and 18 females (60%), with an average age of (43.5 ± 5.2) years; the 30 participants in the healthy control group included 16 males (53.3%) and 14 females (46.7%), with an average age of (40.1 ± 4.7) years. As shown in Table 1, there was no significant difference in sex (t = 0.613, *p* = 0.367) and age (t = 0.173, *p* = 0.467) between the two groups. 

**Figure 2:**
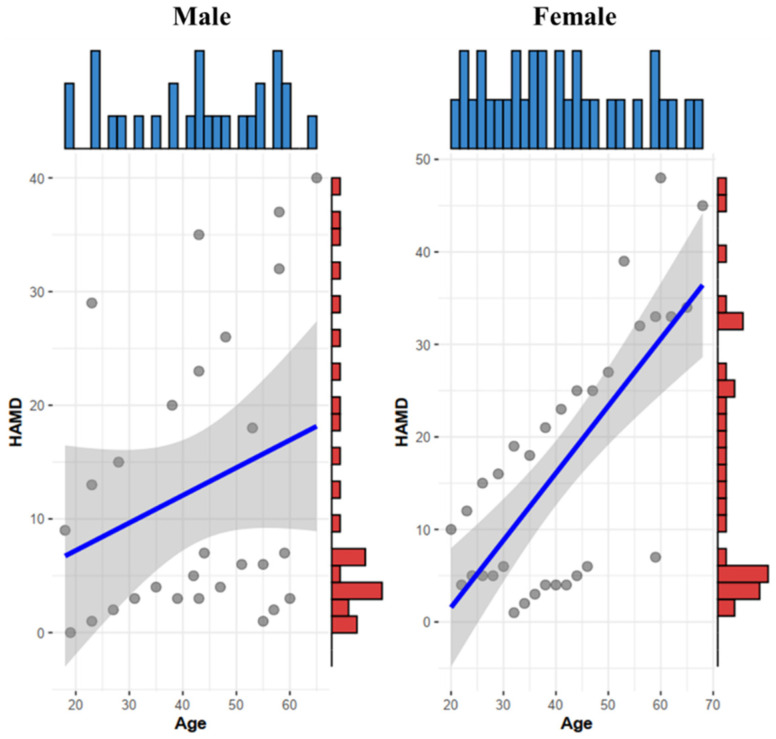
Male and female age and HAMD-24 score distribution chart.

The distribution of HAMD-24 scores in the patient group ranged from 9 to 48 points. A total of 10 patients with a total score of 8–20 points, which was mild depression; 15 patients with a total score of 20–35, which were mild to moderate depression. There were 5 patients with a total score of >35 points, which meant severe depression. The scores of HAMD-24 in the control group were all <8 points, showing a non-normal distribution, and the median (25% and 75%) points were 3 (0, 5). Figure 2 shows the distribution of male and female age and HAMD-24 scores.

### 3.2. Saliva Cortisol

**Figure 3:**
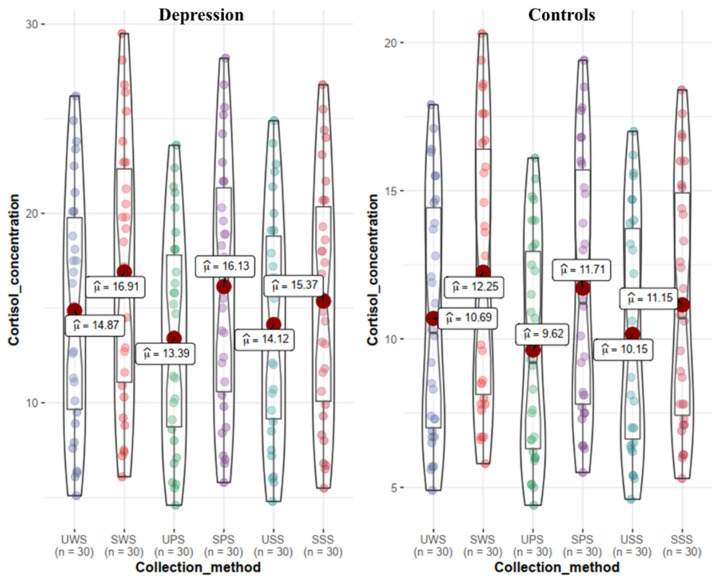
Saliva cortisol levels in the patient group and the control group.

Additionally, the patient group had greater saliva cortisol levels (average levels) than the control group in all six saliva samples (as shown in Table 2), with the highest level of SWS cortisol and the lowest level of UPS cortisol, as illustrated in Figure 3.

The frequency distribution of saliva cortisol levels in the two groups showed that the saliva cortisol levels in the patient group showed a non-normal distribution, and the median (25% and 75%) of different saliva collection methods were different, and the overall distribution range was 6.5–29.4 nmol/L. The saliva cortisol level of the control group showed a non-normal distribution, as shown in Figure 3. The distribution range was 4.8–20.2 nmol/L. Using the Wilcoxon rank sum test to compare the saliva cortisol levels of the two groups, the saliva cortisol level of the patient group was significantly higher than that of the control group, and the difference was statistically significant (*p* < 0.001). The higher the saliva cortisol level, the higher the HAMD-24 score (r = 0.812, *p* < 0.001). There is a slight correlation between cortisol level and age (r = 0.353, *p* = 0.017). There was a slight correlation between cortisol level and sex (*p* = 0.031).

### 3.3. Blood and Saliva Cortisol Correlation

**Figure 4:**
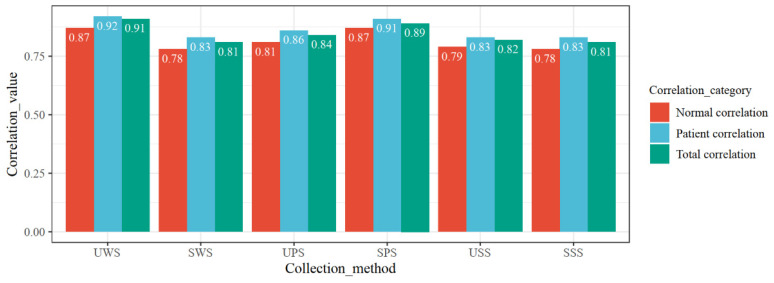
Correlation between six collection methods and blood cortisol.

In this section, six methods (UWS, SWS, USS, SSS, UPS, and SPS) were used for the collection of saliva. The correlation between each saliva sample and blood cortisol was analyzed. Figure 4 shows the correlation between the six saliva collection methods and blood cortisol. It can be seen that the saliva cortisol level obtained by the six saliva collection methods has a very strong correlation with blood cortisol. The UWS was the closest to the blood cortisol level (r = 0.91). It can also be found that the cortisol level of the patient group was correlated with the blood cortisol level, which was higher than that of the control group. Moreover, there was no significant correlation between the irritating saliva collection method and the nonirritating collection method.

### 3.4. Validation of Diagnostic Performance by ROC Curve

**Figure 5:**
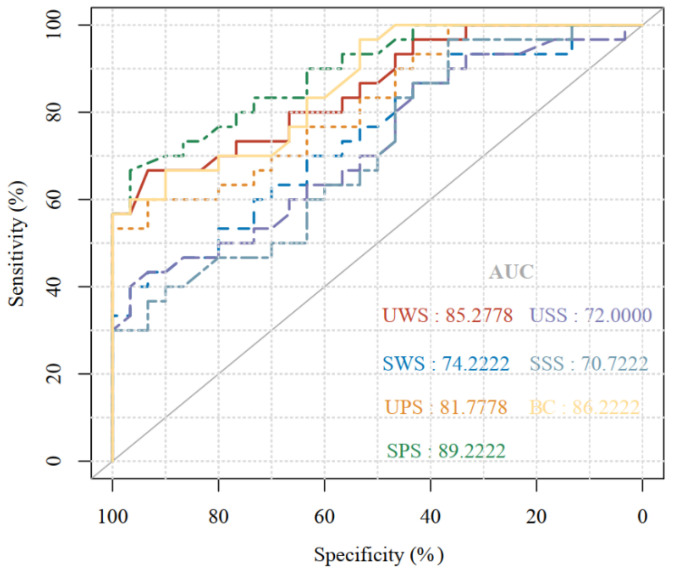
The ROC curve of cortisol in the diagnosis of depression.

According to the ROC curve, the best cut-off value of SPS cortisol level for diagnosing depression was 15.9 nmol/L, with the highest sensitivity and specificity, which were 66.66% and 96.66%, respectively, and the area under the curve (AUC) = 0.89. Next was blood cortisol, with an AUC of 0.86. UWS ranked third with an AUC of 0.85, as shown in Figure 5. In addition, in our study, we found the increased prevalence of depression was related to saliva cortisol ≥ 15.9 nmol/L, the AUC reached 0.75, and the diagnostic performance was classified as good.

## 4. Discussion

Early detection of depression is crucial because only an early diagnosis can provide long-term symptom alleviation [34]. As a result, strategies for identifying the illness in the early stages are badly needed [35,36]. Six saliva collection methods were employed in this study on 30 healthy controls and 30 depressed patients in the morning to determine the ideal saliva collection method. The saliva cortisol levels of these participants were measured using an enzyme-linked immunosorbent assay. We needed to be able to detect saliva cortisol levels using a simple and reliable method. This study also found that the effectiveness of saliva as a diagnostic biological fluid was dependent on the consistency of collection procedures in order to deliver the most accurate and helpful results. The methods used to collect saliva have a considerable impact on cortisol levels and correlation. As a result, standardizing a saliva collection process is crucial for mitigating the impact of variability in saliva composition within and between individuals.

Saliva is frequently misunderstood as a single fluid [37]; instead, saliva is typically divided into single gland saliva and mixed saliva. Parotid saliva, submandibular saliva, and sublingual saliva are examples of single gland saliva [38,39]. The proportionate contribution of different glands to the total saliva sample varies depending on the collection method, level of stimulation, age, and even time of day [40]. Because saliva secretion varies, different methodologies may be required for researching its components or their potential relevance as markers of specific physiological states [41]. Although there is a substantial body of literature on the diagnostic potential of saliva, there is no standardized method for obtaining saliva samples. Different sampling methods are frequently used in different studies, and many studies do not or rarely describe patient preparation or sampling procedures [42,43]. Furthermore, without a complete clinical assessment, participants’ characteristics are frequently insufficient. The majority of saliva cortisol research publications concentrate on analyzing the whole saliva since it is easily acquired by spitting it into a test tube or letting it flow from the mouth [44,45]. Few people are aware of ductal saliva, which is produced by several salivary glands. Furthermore, a cohort with meticulous characterization and clinical assessment was used to compare the cortisol expression of whole saliva and glandular saliva. The results show that different collection procedures produce significant disparities in saliva cortisol snapshots [46]. The findings of this study also show that different saliva collecting procedures produce significant changes in snapshots of saliva biomarkers for depression.

Alternative saliva collection methods would be appropriate for collecting saliva in a clinical situation. It was critical that we could detect saliva cortisol levels using a simple and reliable procedure. We measured saliva cortisol levels using six saliva collection methods in this study: UWS, SWS, USS, SSS, SPS, and UPS. Different saliva collection procedures have a significant impact on cortisol levels and correlation. Second, while the unstimulated approach of directly collecting saliva is practical and easy for patients to accept, the amount of saliva collected is insufficient to meet the needs of detection [47]. Because the effect of stimulation methods on salivary cortisol is unknown, we conducted the first study to compare six different saliva collection methods and investigate the relationship between saliva cortisol and blood cortisol level. The main conclusions derived from this work were summarized as follows: The UWS cortisol level was strongly associated with the blood cortisol level, but the SPS cortisol level can be better used to predict depression. In participants with depression and without depression, there was a slight correlation between cortisol levels and age, and females had a higher prevalence of depression than men. It was found that the saliva cortisol level of depressed patients was higher than that of healthy controls. The higher the saliva cortisol level, the higher the HAMD-24 score. It has been proved that saliva cortisol testing as an auxiliary method for the diagnosis of depression can help identify patients at risk of depression for early prevention strategies. 

The HAMD-24 score of the patient group was normally distributed, with a mean ± standard deviation of (25 ± 10) points and a distribution range of 9 to 48 points. The depression patient group covered mild, moderate, and severe conditions. Among the 30 people selected in the patient group, 18 were female, and 12 were male, which was consistent with the higher incidence of depression in females than in males [48,49]. The average age of the patient group was (43.5 ± 5.2) years, which was also in line with the higher incidence of depression and menopause [50,51]. This may be because females show greater activation of the HPA axis than males, and the loss of estrogen during menopause shows the greatest HPA axis dysregulation [52,53]. The distribution of HAMD-24 scores in the control group ranged from 0 to 7, and none of them had depressive symptoms. The higher the saliva cortisol level, the higher of HAMD-24 score, indicating that the saliva cortisol level in the morning can reflect the severity of depression in people with depression, which was consistent with conclusions of previous studies [54,55]. This study found that the saliva cortisol level of females was different from that of males, and the difference was statistically significant (*p* < 0.05), suggesting that the difference in saliva cortisol may be related to sex in patients with depression. The saliva cortisol level in the group of female patients with depression may be higher than that in males, and it needs to be further verified by multiple trials. In the control group, there was no difference between the saliva cortisol levels of females and males, and the difference was not statistically significant (*p* > 0.05), which was inconsistent with the results reported by some larger cortisol studies. It is considered that the sample of participants in this study is small, and the saliva cortisol difference that may exist between the sexes has not been found.

Cortisol is a hormone related to the HPA axis [56]. It has a strong circadian rhythm. Its level reaches its highest peak within 1 h in the morning and then decreases rapidly [57,58], so this study strictly followed the collection time during the specimen collection process to ensure the accuracy of data. This study compared six different saliva collection methods and explored the relationship between saliva cortisol and blood cortisol levels. It was found that the unstimulated whole saliva cortisol level was most correlated with the blood cortisol level, which can accurately reflect the level of blood cortisol. It further suggests that saliva cortisol can be used as a measure of stress response to assist in the diagnosis of depression.

At the same time, this study found that the level of nonirritating saliva cortisol was continuously lower than that of stimulating saliva cortisol, which is consistent with the results reported in previous studies [59,60,61]. Poll [62] also recently demonstrated that the collection method affects the measurement accuracy of cortisol in saliva. The ROC analysis method combines sensitivity and specificity for analysis and is an ideal method for the comprehensive and accurate evaluation of diagnostic tests [63,64]. The ROC analysis method was used to evaluate the diagnostic value of saliva cortisol detection for depression, and the calculated AUC was 0.765 (95% confidence interval: 0.679–0.838). According to the evaluation criteria of Swets [65], when the AUC = 0.5, the diagnosis is completely ineffective; when the AUC is less than 0.5, it is not in line with the actual situation; it is generally believed that 0.5 < AUC ≤ 0.7 indicates low diagnostic value. It is only for reference in practical applications; 0.7 < AUC ≤ 0.9 indicates a certain degree of accuracy and can be used in clinical diagnosis, with a medium diagnostic value; AUC > 0.9 indicates a relatively high diagnostic value, which can be used in clinical diagnosis as an important diagnostic basis [66,67]. In the results of this study, the AUC was 0.89, close to 0.9, so we think that saliva cortisol testing can be considered an auxiliary method for the clinical diagnosis of depression, and its diagnostic value is moderate. In addition, the collection of saliva cortisol in this study allows for non-invasive collection at regular intervals, which can be stably collected for several days before the experiment, so that the HPA axis in the free state can be effectively evaluated [68,69]. The assessment of cortisol in saliva has proven effective and reliable in reflecting the respective unbound hormones in the blood [70,71]. Therefore, in this study, we assessed the level of cortisol through saliva samples. In addition, compared with the enzyme-linked immunosorbent assay, saliva cortisol is determined by chemiluminescence immunoassay, which has higher sensitivity and specificity [72].

However, this study has shortcomings. First of all, it is difficult to determine the causal relationship between only one assessment of saliva cortisol and current depression symptoms. Secondly, this study lacks a concurrent evaluation of the HPA axis function, and there is no information about saliva gene expression. In addition, the exclusion criteria do not include corticosteroid therapy for somatic diseases that may affect the HPA axis function. Finally, saliva cortisol secretion presents a circadian rhythm. This study used morning saliva cortisol for research and analysis. Although previous studies support that the morning cortisol level is the highest and the best time for measurement, it is necessary to perform multiple time-point research so as to fully understand the role of saliva cortisol in the diagnosis of depression. Due to the limited number of data points, further research is necessary, including validity testing, retesting, and multi-factorial testing, even if this study offers the best performance for the early detection of depressed patients. Finally, there are still many issues in this study that need to be resolved and further investigated. For instance, the proximity of the submandibular and sublingual glands makes it challenging to categorically distinguish the saliva from both glands. For this reason, saliva was taken from both glands in the current investigation. The distinction between sublingual and submandibular saliva is another area that requires more investigation. In order to make the diagnosis of depression in saliva more accurate, numerous risk variables (such as living environment, work environment, etc. [73]) should also be taken into consideration.

## 5. Conclusions

This study found that the levels of cortisol in saliva are highly correlated with those found in the blood. Moreover, the significant difference in saliva cortisol level depends on the saliva collection method, the level of UWS cortisol was most correlated with blood level; however, the SPS cortisol can better predict depression. In addition, this study found that the saliva cortisol level of the patients with depression was significantly higher than the patients without depression. Moreover, the correlation between the cortisol level and the HAMD-24 score was highly significant. The higher the saliva cortisol level, the higher the HAMD-24 score. Finally, it was found that cortisol level has a slight correlation with age and sex. This study demonstrated that early morning saliva cortisol has excellent diagnostic characteristics and, as such, is a robust, convenient test for screening and diagnosis of depression, which can help people with depression be aware of their negative thoughts and early prevention of them.

## Figures and Tables

**Table 1:** Sample characteristics of the studied groups.

Sample Characteristics	Depression Group	Healthy Controls	*p*
Age	43.5 ± 5.2	40.1 ± 4.7	0.467
HAMD-24 scores	25.7 ± 10.3	3.9 ± 1.8	<0.001

**Table 2:** The average levels of saliva cortisol for each group (depressed patient/control and stimulated/unstimulated).

Cortisol Levels (nmol/L)	UWS	SWS	UPS	SPS	USS	SSS
Patients (N = 30)	14.87 ± 6.22	16.91 ± 6.91 **	13.39 ± 5.60	16.13 ± 6.61	14.12 ± 5.91	15.37 ± 6.29
Controls (N = 30)	10.69 ± 4.07	12.25 ± 4.53	9.62 ± 3.67 *	11.71 ± 4.33	10.15 ± 3.87	11.15 ± 4.11

** Maximum level of saliva cortisol. * Minimum level saliva cortisol.

## Data Availability

The study did not report any data.
